# Percutaneous Ilioilial Fixator Versus Percutaneous Iliosacral Screw in Managing Unstable Sacral Fractures: A Prospective Randomised Controlled Study

**DOI:** 10.7759/cureus.54358

**Published:** 2024-02-17

**Authors:** Mohamed Shaalan, El Zaher H El Zaher, Ossama M Farag, Ahmad G Abdallatif, Ahmed M Sallam

**Affiliations:** 1 Trauma and Orthopaedics, Worcester Royal Hospital, Worcester, GBR; 2 Orthopaedic Surgery, Ain Shams University, Cairo, EGY

**Keywords:** prospective study, iliosacral screw, ilioilial fixator, unstable sacral fracture, pelvic fracture

## Abstract

Introduction: Unstable sacral fractures with pelvic fractures are challenging to both surgeons and patients, particularly in the immediate post-injury phase and later when definitive fixation is undertaken. Percutaneous iliosacral screw fixation is widely regarded as the gold standard treatment for unstable sacral fractures without spinopelvic dissociation. Closed reduction and percutaneous fixation using iliosacral screws for sacral fractures provide early stabilisation without the need for extensive surgical exposure, thereby mitigating major complications associated with open surgical procedures. A new technique for stabilising unstable sacral fractures is the minimally invasive ilioilial fixator, also called a transiliac internal fixator (TIIF), which has gained more attention for its ability to address challenges associated with sacroiliac screw fixation.

The objective of this study is to compare the functional, radiological, and surgical outcomes between the percutaneous iliosacral screw and the ilioilial fixator.

Methods: A total of 51 patients with sacral fracture injuries sustained between August 2019 and November 2021 were included in this study, with 25 patients in Group A and 26 patients in Group B. Patient randomization was done using computer-generated randomization facilitated by Random Allocation Software (Mahmood Saghaei, Isfahan, Iran). All patients underwent the chosen intervention within 10 days of the trauma. Patients had follow-up at two weeks, six weeks, and 12 months post-treatment. The results of fixation were evaluated radiologically based on the Matta and Tornetta grading system and clinically using the Majeed pelvic scoring system. Complications were detected in both groups during follow-up visits.

Results: The study found no statistically significant differences between the two patient groups in terms of final clinical assessment (p=0.79), radiological assessment (p=0.78), or the need for another operation (p=1.0). Moreover, there were no statistically significant differences between the groups with respect to complication rates (p=0.63) or the time of union (p=0.14). No differences were noted in terms of intraoperative blood loss (p=0.93) or operative time (p=0.34) but for longer incision length in the ilioilial fixator group (p<0.001) and an increased risk of intraoperative radiation exposure in the iliosacral screw group (p<00.1).

Discussion: Although the iliosacral screw is considered a gold standard for unstable sacral fracture, a TIIF is a good alternative with a very satisfactory outcome.

Conclusion: Although the iliosacral screw still remains the gold standard for the management of sacral fractures, the ilioilial fixator emerges as a good alternative with comparable functional and radiological outcomes.

## Introduction

Despite the high incidence of unstable sacral fractures with pelvic injuries, there is no single management algorithm suitable for all sacral fractures. Nonetheless, surgical treatment is regarded as the gold standard in definitive management [1].

The primary objectives of operative intervention are to reduce fracture fragments, protect neurological structures, and provide adequate stability, facilitating early mobilisation [2].

Many surgical techniques have been described for the internal fixation of the unstable sacral fracture associated with pelvic fractures. Stabilisation through the use of an iliosacral screw is considered a gold standard technique for sacral fracture fixation. Recently, other techniques of stabilisation have gained more attention, especially in cases where an iliosacral screw is deemed unsuitable [3].

Percutaneous iliosacral screw fixation has potential advantages, including “reduced blood loss and soft tissue stripping,” along with lower postoperative infection rates compared to alternate procedures. Nevertheless, iliosacral screw fixation needs thorough preparation, meticulous closed reduction, and adequate surgical skills to prevent injuries during the screw fixation process. Additionally, iliosacral screw fixation encounters many challenges when dealing with sacral dysmorphism, obesity, and challenges relating to inadequate intraoperative images [4].

A relatively new technique for stabilising the posterior pelvic ring is the placement of pedicle screws in both posterior iliac crests, combined with a transverse rod crossing the midline of the posterior sacrum. This minimally invasive device is often called a transiliac internal fixator (TIIF) [5].

The TIIF fixation technique has several advantages, including its ability to overcome the challenges posed by the iliosacral screw fixation technique. It has better reduction control, reduces the risk of neurological complications, and can be performed without the need for intraoperative images [5].

The aim of this study is to conduct a comparative analysis between percutaneous iliosacral screw fixation and ilioilial fixation in the context of unstable sacral fracture fixation stabilisation. The assessment includes various aspects such as operative time, the need for intraoperative fluoroscopy, blood loss and related transfusions, infection rates, occurrences of neurovascular complications, hardware failures, screw misplacements, clinical and radiological outcomes, revision rates, and the rate of non-union. We excluded fractures with spinopelvic dissociation and neurological injuries from this analysis.

## Materials and methods

Ethical approval and setting

Approval from the Research and Ethics Committee of Ain Shams University Hospital (approval number: FMASU M D 33/2020) was obtained. Fifty-one patients with unstable sacral fractures were recruited between August 2019 and November 2021.

Inclusion and exclusion criteria

Inclusion criteria include acute unstable sacral fractures occurring in less than 10 days (amenable to closed reduction techniques) and an age range of 16 to 60 years. The exclusion criteria for the study included patients with spinopelvic dissociation or those associated with neurological injuries. All patients were monitored for a minimum of one year.

The primary mode of trauma was mainly motor vehicle accidents (68%), although other mechanisms of injury were also identified, including falls from heights and pedestrian accidents (16% and 12%, respectively). One case involved an injury from a heavy object falling onto the pelvis.

Sample size calculation and statistical power

Fifty-one patients with unstable sacral fractures were recruited between August 2019 and November 2021. The first group included 25 patients, while the second group included 26 patients. The sample size was found to have 80% statistical power.

Randomization, allocation, and blinding

Randomization of patients was conducted through the use of computer-generated randomization, which is facilitated by the Random Allocation Software (Mahmood Saghaei, Isfahan, Iran), resulting in two groups: Group A, comprising 25 patients treated with iliosacral screws, and Group B, 26 patients treated with the ilioilial fixator. The confirmation of sacral fracture instability was established using preoperative radiography (X-rays and CT scans).

It is worth noting that all fractures were part of traumatic pelvic fractures. The study was designed as double-blind, with patients providing consent for both techniques. The surgeon gets to know the chosen technique only on the day of the operation, as indicated by a closed envelope.

Surgical methods/techniques

In the first group, nine out of 25 patients (36%) had only iliosacral screw fixation, while the rest of the patients underwent additional fixation methods, including an external fixator (9/25), INFIX (2/25), and a symphyseal plate (5/25). In the second group, 17 out of 26 patients (65%) had solely ilioilial fixation, while the rest had additional types of fixation, including an external fixator (4/26), INFIX (3/26), and a symphyseal plate (2/26).

**Figure 1:**
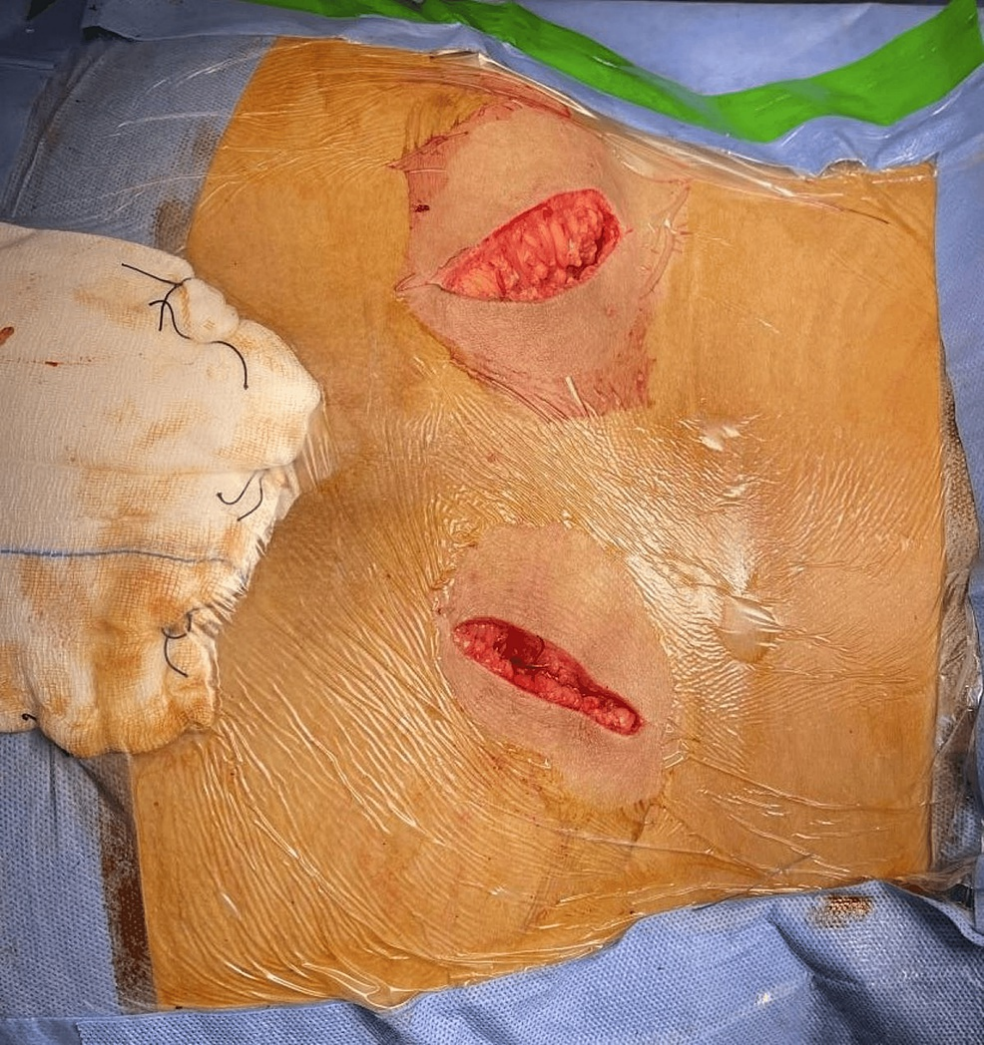
Minimally invasive two curvilinear incisions over the PSIS taken during the procedure PSIS: posterior superior iliac spine Image Credit: First Author

All patients managed by the iliosacral screw underwent surgical techniques that depended on closed reduction with percutaneous fixation under the guidance of C-arm control [6]. Furthermore, all patients managed with TIIF underwent minimally invasive 2-3 cm curvilinear incisions over the posterior superior iliac spines (PSIS). The procedure involved exposing the PSIS and applying a rongeur bite to the medial aspect of the PSIS and the area just below it (Figure 1).

**Figure 2:**
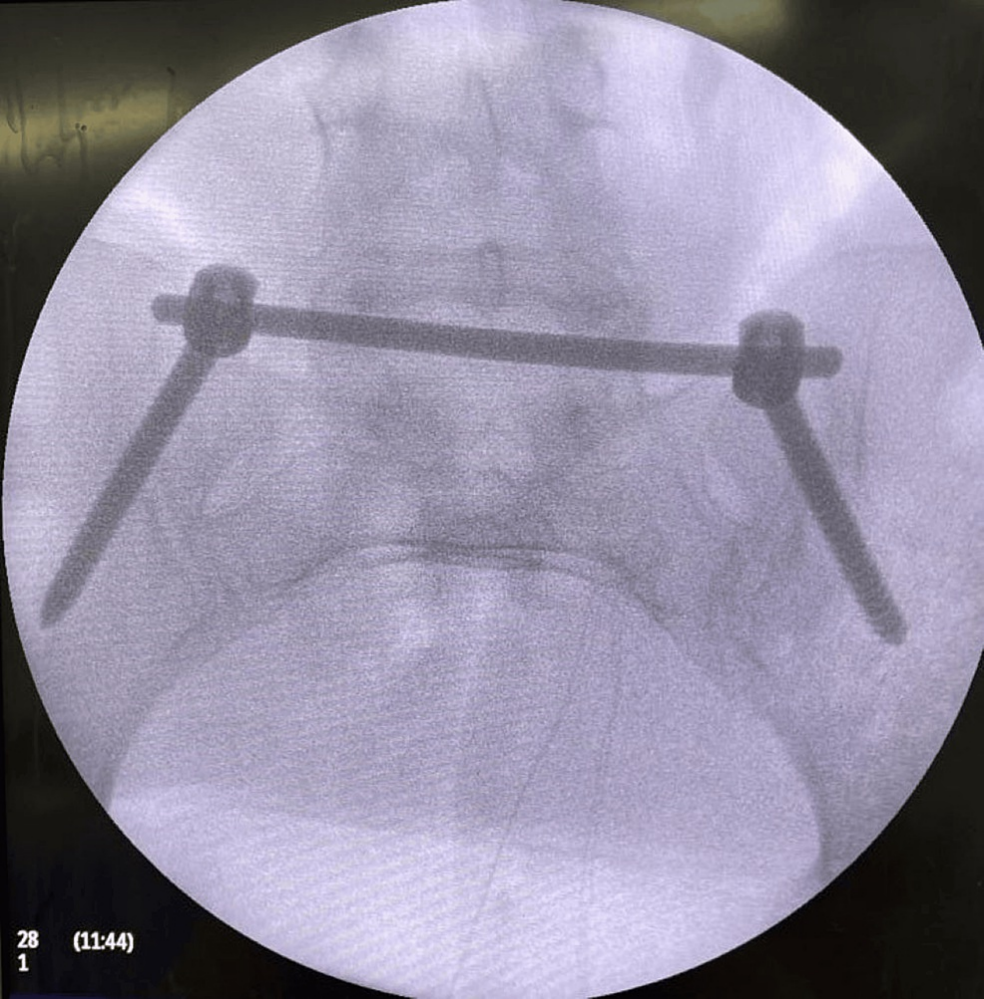
Intraoperative image of the pelvic inlet view showing two polyaxial pedicle screws and connecting rod inserted into the iliac bone toward the greater trochanter Image Credit: First Author

Two titanium polyaxial or monoaxial pedicle screws, measuring 55 to 80 mm in length and 7 mm in thickness, were applied, with two screws placed on each iliac bone. These screws were directed towards each ipsilateral greater trochanter, and, in most cases, the procedure was carried out without the aid of a fluoroscope (Figure 2).

A deep tunnel was created to percutaneously connect the two screw insertion sites, passing deep to the paravertebral muscles for the passage of the two interconnecting rods. The rod was then positioned between the two parallel iliac screw heads, and the final intraoperative picture was taken [7].

After the surgery, the postoperative plan entailed passive hip movement while in bed for two weeks, followed by an assisted partial weight-bearing protocol from the second to the sixth week for both groups, with passive and active-assisted hip movement. Then, full weight-bearing and a physiotherapy programme were initiated to strengthen abductors and quadriceps muscles after 12 weeks, contingent on pain tolerance.

Data management

Preoperative data were collected with regard to patient age and sex. All our patients in the study ranged in age from 16 to 52 years old. Thirty-four males and 17 females were included in the study. Both age and sex were non-significant, and the different demographics did not affect our results. Motor car accidents represent the most common cause of sacral fracture in our study (Table 1).

**Table 1:** Mode of trauma in relation to the fixation principles *Chi-square test, FE: Fisher exact, MCA: motor car accident, N: number

Mode of trauma	Fixation principle				X^2*^	p-value
	Iliosacral screw		Ilioilial fixator			
	N	%	N	%		
MCA	17	68.0%	17	65.4%	2.08 FE	0.69
Falling from height	4	16.0%	3	11.5%		
Pedestrian accident	3	12.0%	6	23.1%		
Heavy object on the pelvis	1	4.0%	0	0%		

**Figure 3:**
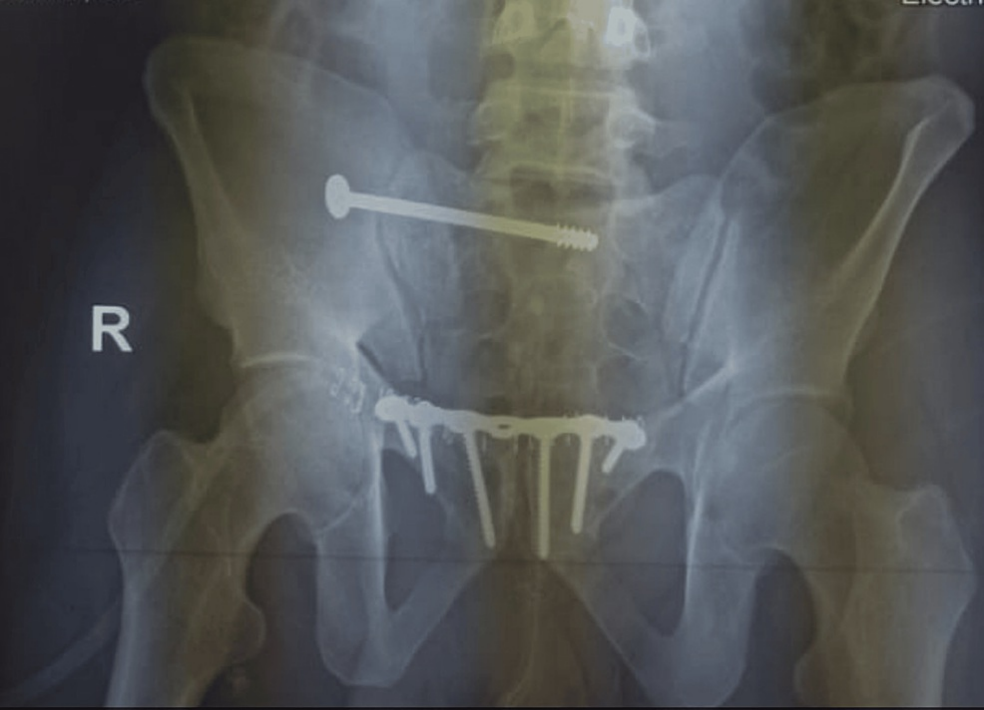
Postoperative X-ray outlet view of the pelvis showing excellent reduction of the sacral fracture using iliosacral screw fixation above the S1 foramen with fixation of the anterior pelvic ring injury using symphysis plating Image Credit: First Author

**Figure 4:**
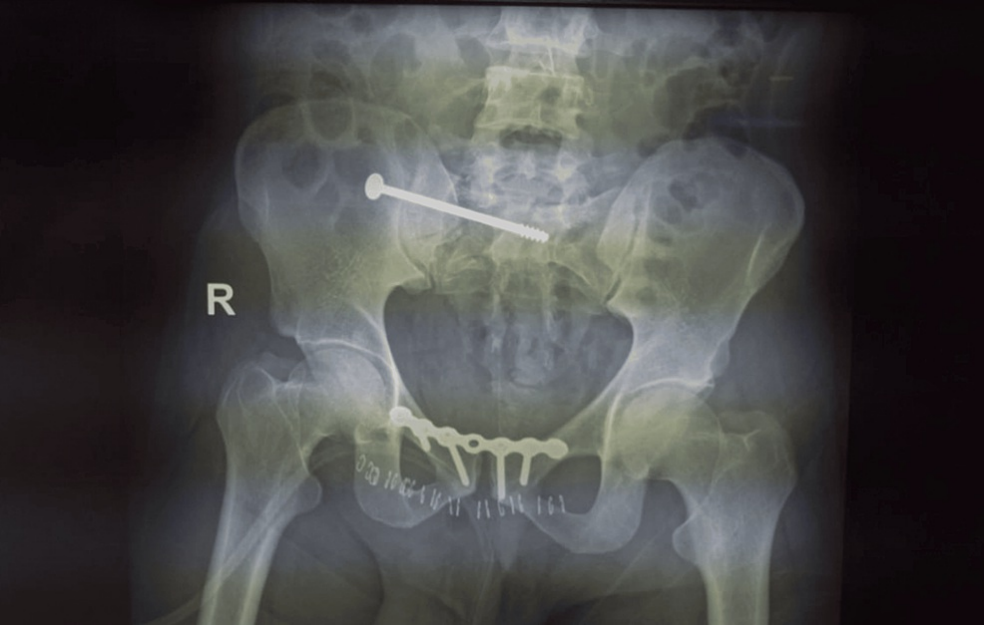
Postoperative X-ray posteroanterior view of the pelvis showing excellent reduction and fixation of the sacral fracture using an iliosacral screw with fixation of the anterior pelvic ring injury using anterior symphysis plating with the good restoration of ring alignment Image Credit: First Author

**Figure 5:**
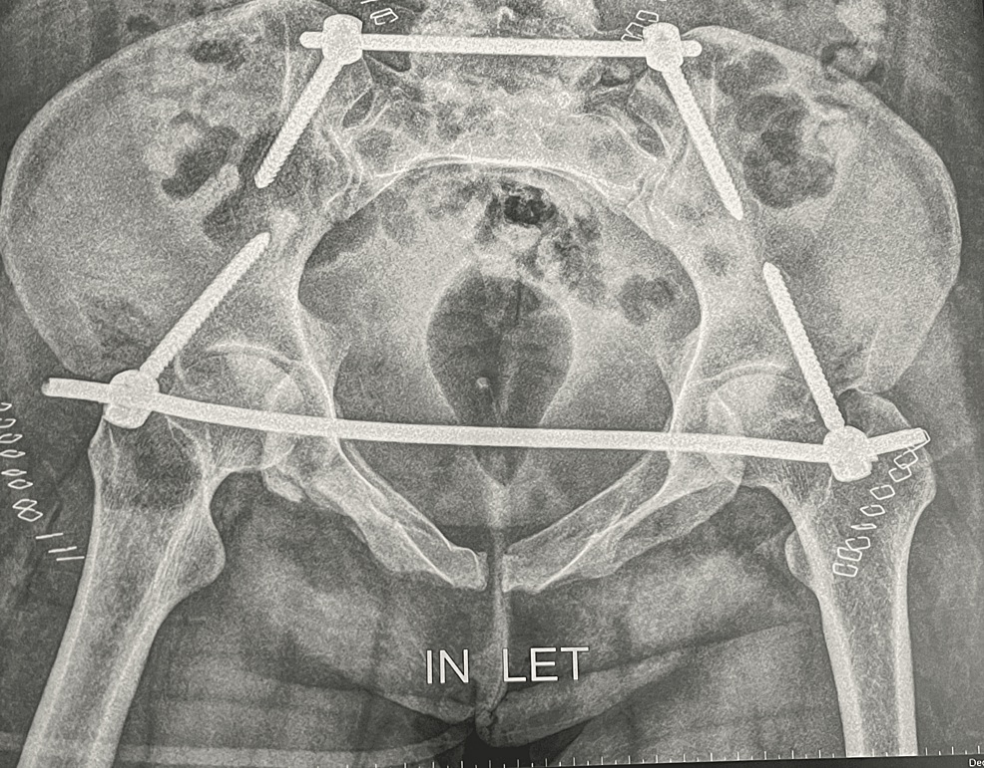
Postoperative X-ray inlet view of the pelvis showing excellent reduction of the sacral fracture with ilioilial fixator and good fixation of the anterior pelvic ring with INFIX with the overall good restoration of ring alignment Image Credit: First Author

After the surgery, postoperative X-ray examinations and neurovascular assessments were conducted the next day (Figures 3-5).

**Figure 6:**
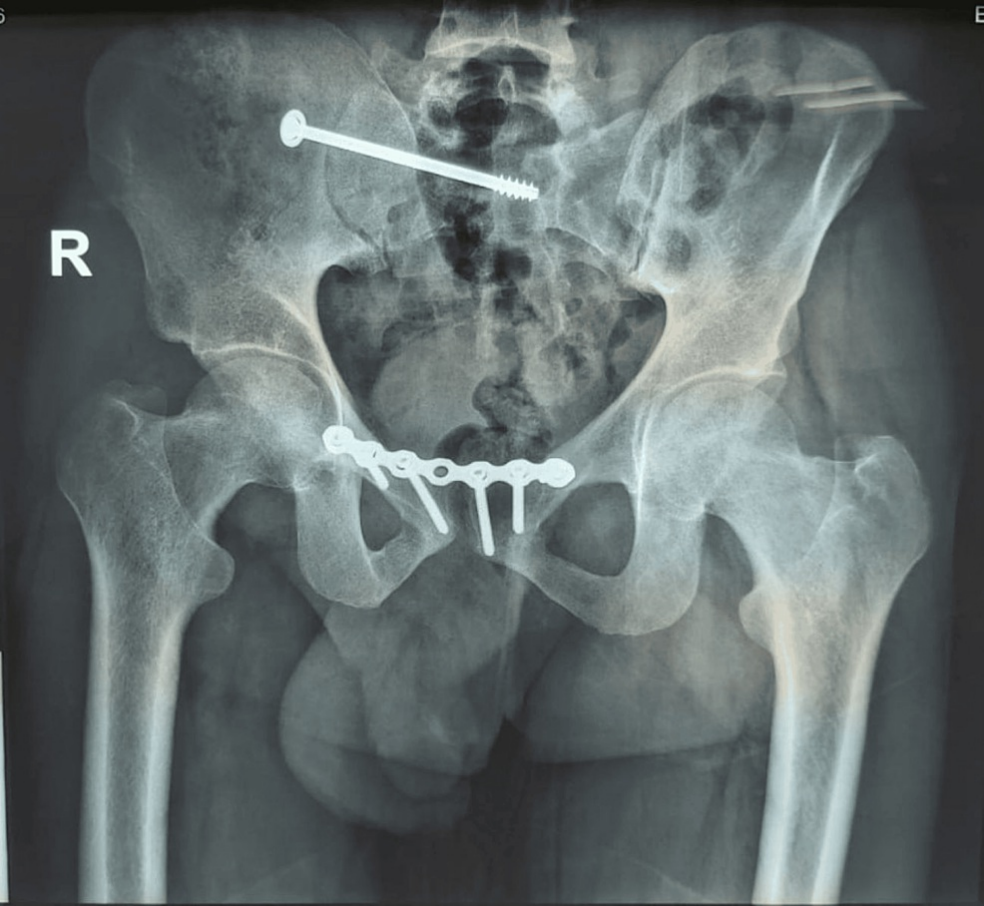
One-year follow-up X-ray posteroanterior view of the pelvis showing complete healing at the sacral fracture fixed with iliosacral screw with stabilization of anterior pelvic ring by symphysial plating Image Credit: First Author

Scheduled follow-up visits were arranged at intervals of two weeks, six weeks, three months, six months, and then one year postoperatively. During these visits, plain X-ray imaging was performed, showing both hips in anteroposterior, inlet, and outlet views (Figure 6).

The acquired X-rays were evaluated using the radiological principles of Matta and Tornetta [8]. Five criteria were evaluated on the postoperative X-ray films, which were included in the measurement for residual posterior displacement, vertical displacement, pubic symphysis translation, sagittal rotation, and gapping of the sacroiliac joint. According to Matta and Tornetta, the results are classified as excellent (<4 mm), good (4-10 mm), fair (10-20 mm), and poor (>20 mm). Furthermore, clinical evaluations were conducted using the Majeed pelvic scoring system during the one-year follow-up visit [9]. In the follow-up visits, patients were assessed for wound challenges, loss of reduction, broken screws, implant failure, leg length discrepancies, postoperative infections, the necessity for another operation, and hardware-related irritations.

Statistical analysis

Data were collected, revised, coded, and entered into SPSS Statistics version 23 (IBM Corp. Released 2015. IBM SPSS Statistics for Windows, Version 23.0. Armonk, NY: IBM Corp.). The quantitative data with parametric distribution were presented as means, standard deviations, and ranges. The qualitative variables were presented as numbers and percentages. The comparison between groups regarding qualitative data was done using the chi-square test. The comparison between two independent groups with quantitative data and a parametric distribution was done using an independent t-test.

## Results

Intraoperatively, operative time, blood loss, incision length, and fluoroscopic time were obtained (Table 2).

**Table 2:** Mean difference of the operative data of the two fixation groups *Student t-test, SD: standard deviation

Variables	Fixation principle				t*	p-value
	Iliosacral screw		Ilioilial fixator			
	Mean	SD	Mean	SD		
Operations time (min)	76.60	32.43	86.65	25.63	0.97	0.34
Incision length (cm)	1.20	0.41	4.85	1.01	17.05	<0.001
Blood loss	125.40	91.68	123.72	73.69	0.09	0.93
Number of intraoperative fluoroscopic	38.08	13.34	9.73	5.63	9.82	<0.001

There is no statistically significant difference between union time and postoperative complications between the iliosacral screw and the ilioilial fixator in managing sacral fractures (Table 3).

**Table 3:** Percentage difference of the outcomes of the two fixation principles SD: standard deviation, *Student t-test, **Chi-square test

Variables		Definitive management				t*	p-value
		Iliosacral screw		Ilioilial fixator			
		Mean	SD	Mean	SD		
Follow-up (months)		14.80	3.16	16.62	3.87	1.83	0.07
Union (months)		4.16	0.75	3.85	0.73	1.52	0.14
		N	%	N	%	X^2**^	p-value
Postoperative complications	No	17	68.0%	16	61.5%	0.23	0.63
	Yes	8	32.0%	10	38.5%		

There was no statistically significant relationship between final clinical assessment, radiological assessment, the need for another operation, and fixation principles (Table 4).

**Table 4:** Postoperative clinical and radiological assessment of the two fixation groups *Student t-test, **Chi-square test, FE: Fisher exact

Variables		Fixation principle				t*	p-value
		Iliosacral screw		Ilioilial fixator			
		Mean	SD	Mean	SD		
Final clinical assessment		82.16	13.43	80.46	10.00	0.51	0.61
		N	%	N	%	X^2**^	p-value
Matta radiological	Excellent	17	86.0%	15	57.7%	0.88 FE	0.78
	Good	5	20.0%	8	30.8%		
	Fair	3	12.0%	3	11.5%		
Final clinical assessment	Excellent	19	76.0%	18	69.2%	1.77 FE	0.79
	Good	4	16.0%	6	23.1%		
	Fair	1	4.0%	2	7.7%		
	Poor	1	4.0%	0	0%		
Need for another operation	No	23	92.0%	23	88.5%	0.18 FE	1.00
	Yes	2	8.0%	3	11.5%		

## Discussion

Our study showed that both the percutaneous ilioilial fixator and percutaneous iliosacral screw fixation are effective modalities for the treatment of sacral fractures without spinopelvic dissociation. We observed no significant differences in perioperative and postoperative assessments, except for the longer incision length required in the ilioilial fixator and the increased risk of intraoperative radiation exposure in the iliosacral screw group.

Despite its widespread acceptance and application, the placement of iliosacral screws remains a persistent challenge, primarily due to various reasons such as abnormal osseous anatomy in the posterior pelvic ring, upper sacral segment dysmorphism, and the influence of obesity [10]. The ilioilial fixator represents a valid, minimally invasive method of sacral fracture fixation. The device can be implemented quickly and easily, has less dependence on fluoroscopy, and is associated with low blood loss, low complication rates, and high union rates. The fixator acts as a suspension bridge structure, such as the sacroiliac joint complex, partly maintaining the integrity of the pelvic ring.

Blood loss

Our study’s observations indicate that there is no significant difference in blood loss between the iliosacral screw and ilioilial fixator groups. Although the mean blood loss was nearly the same in both groups, it is worth noting that the calculated blood loss is overestimated by the simultaneous anterior approach and fixation methods (symphyseal plating, INFIX, and external pelvic fixator). In nine cases, the iliosacral screw was used as a standalone procedure, resulting in a mean blood loss of 33 ml, while the ilioilial fixator was the sole method of fixation in pelvic injury cases in 17 cases, leading to a mean blood loss of 77.85 ml. It should be emphasised that iliosacral screw fixation, when used in isolation, typically results in trivial blood loss. Both techniques continue to be considered minimally invasive.

Two retrospective studies analysing posterior pelvic injuries treated with the ilioilial fixator by Bi et al. and Wang et al. reported lower blood loss results, measuring 43.42±4.90 ml and 46.7±4.9 ml, respectively. These figures exclude associated procedures involving anterior pelvic fixation [11,12]. In another study, Korovessis et al. used dual ilioilial fixators and reported blood loss of less than 100 ml [13].

Incision length and postoperative infection

There is a statistically significant difference in incision length between the two groups, as the mean length of incision in the iliosacral screw and the ilioilial fixator is 1.2 cm and 4.8 cm, respectively. The ilioilial incision depends on the cumulative experience with the technique, and increasing the learning curve decreases the length. Repeated dressing and antibiotics managed three cases with superficial infection, while only one case needed debridement due to deep infection and wound dehiscence, and there was no wound complication in the iliosacral group. Clinically, wounds in each group heal well.

Operative time

Statistically, there is no difference in operative time between both groups (the time spent turning the patient from the prone to supine position was not included in the operation time in the ilioilial group). Clinically, it is observed that the ilioilial fixator is more time-consuming, especially if it is associated with an anterior approach and another fixation is needed. The process of changing the patient’s position from prone to supine, along with the necessary sterilisation and disinfection procedures, will prolong the duration of the overall procedure.

There is no consensus on the long-term outcomes of adding anterior stabilisation to posterior fixation in pelvic fractures. Therefore, it may be encouraging to many surgeons to opt for posterior fixation using the ilioilial fixator alone if the achieved reduction is accepted, rather than changing the position and starting from the first for anterior stabilisation [14].

In our study, the iliosacral screw was used as a standalone procedure without anterior fixation in nine cases (mean operative time: 46.25 minutes). In the study conducted by Shuler et al., the mean operative time was 52 minutes for 35% of the patients who underwent percutaneous screw fixation without additional procedures [15]. In a comparative study by Grossterlinden et al., it was suggested that surgeons experience increased accuracy, decreased operative time (with a mean operative time per screw of 29.4 minutes), and reduced radiation exposure in iliosacral screw procedures. It was further emphasised that this should only be performed under highly experienced surgical supervision [16].

In our study, the ilioilial fixator was used as the only method of fixation in 17 cases (mean operative time: 58.52 minutes). In a retrospective study conducted by Tempelaere et al., the mean surgery time was 45 minutes [17]. In another study by Korovessis et al., the mean operative time for posterior pelvic ring injuries was 76 minutes (ranging from 68 to 80 minutes) for the dual fixator procedure [13].

Fluoroscopy and radiation exposure

There is a statistically significant relationship between the number of intraoperative fluoroscopic and fixation principles (p<0.001) and the higher risk of radiation exposure in the iliosacral screw group. The iliosacral screw procedure is completely C-arm-dependent. Due to the limitations of 2D-fluoroscopic images, a repetitive change of the C-arm projection (90° to another) is mandatory to visualise the guide wire position in all three dimensions during drilling. Furthermore, the experience of the surgeon in pelvic surgery is an important factor, which may further influence these parameters. One of the most important advantages of the ilioilial fixator is that there is less dependence on the fluoroscopy, and the procedure can be done totally without an image intensifier depending on the anatomical landmark, making the fixator an applicable intervention in emergencies, technical challenges, or poor visualisation due to obesity or pelvic gases [17,18].

Union

Our study did not reveal any statistically or clinically significant differences in union between the two groups. We had no cases of non-union in both groups. Both fixation methods are minimally invasive, preserving the fracture hematoma with less soft tissue dissection and similar biomechanical properties, so it is expected to get similar union results. Sacral non-union after closed manipulative reduction and percutaneous fixation of a displaced sacral fracture is rare [3].

Khaled et al. [19] and Kim et al. [20] showed similar results of a 100% union rate in cases managed with iliosacral screws for posterior pelvic ring injuries. Liuzza et al. had only one case of sacral fracture non-union out of 19 cases managed by the iliosacral screws [2].

Analysed data from 168 cases presented in a systematic review of ilioilial fixators for posterior pelvic ring injuries by Muller et al. showed a high union rate with no reported cases of non-union [21]. similar union rate presented in another study [17,22,23].

Postoperative complications

Although there is no statistically significant difference in the rate of postoperative complications between both groups, there is marked clinical relevance regarding the nature and effect of these complications on the functional outcome (Table 5).

**Table 5:** Percentage of postoperative complications of iliosacral screws vs. Ilioilial fixator in sacral fracture fixation

Postoperative complication	Iliosacral screw	Percentage from the total	Ilioilial fixator	Percentage from the total
Number of cases with complications	8	32%	10	38%
Screw cutout	5	20%	0	0
Residual fracture displacement	2	8%	0	0
Leg length discrepancy	4	16%	4	15%
Hardware irritation	0	0	2	7.6%
Non-union or mal union	0	0	0	0
L5 (lumber nerve root 5) injury	3	12%	0	0
Superficial infection	0	0	3	11.5%
Revision surgery	2	8%	0	0
Need for implant removal	0	0	2	7.6%
Deep infection	0	0	1	3.8%

In each group, four patients had leg length discrepancies due to pelvic-related causes, ranging from 3 mm to 20 mm. The LLD of less than 2 cm was managed with the use of shoe elevation.

In the ilioilial fixator group, three cases of superficial infection were successfully treated by the use of culture and sensitivity, appropriate antibiotic therapy, and wound dressing. However, there was one case with deep infection and wound dehiscence, necessitating surgical debridement. Additionally, only two cases in the ilioilial fixator group underwent fixator removal for hardware irritation. Most of the postoperative complications in both groups in our study were found to be correctable, resulting in little impact on functional outcomes, with the exception of screw malposition, especially when it resulted in neurological injury.

In our study, 20% (5/25) of sacral fractures exhibited screw cutout or loosening, but only two cases underwent revision (8%). The percentage of postoperative neurological injury was 12% (3/25), but only one case had a permanent insult (foot drop). The other two cases spontaneously recovered within three months, while one case underwent screw removal and recovery. Notably, Grossterlinden et al. reported a 15% rate of misplaced screws in OTA-type 61-C fractures in the fluoroscopic-controlled group [16].

In another study involving 131 patients, Zwingmann et al. reported a rate of 36% for malposition screws [24]. This is in contrast to the findings of Osterhoff et al. (malposition was only evaluated on conventional pelvic radiographs), who found misplaced screws in 4.82% (4/83) of cases [25]. In our study, postoperative CT scans were conducted only in cases involving postoperative neurological injury or revision surgery. Screw malposition has been reported to occur between 3% and 17%, with the risk of neurological injury ranging from 0% to 8% in patients. The wide range of malposition rates has been reported to be mainly linked to differences in the definition of malposition. Some studies define malposition as any screw penetration of the bony cortices, regardless of the direction of the penetration, while in other studies, screws are frequently considered “malpositioned” only when revision surgery is required [24,26,27].

In our study, all cases of screw cutout were classified as type C fractures according to the Tile classification system and were fixed with a single iliosacral screw. It may be explained by the sufficient stability provided by a single iliosacral screw in the management of a vertical sacral fracture or a type C pelvic fracture [16].

There are no cases of postoperative neurological injuries, screw cutout or loosening, or revision in the ilioilial group. Additionally, our study did not report any instances of mortality, symptomatic deep venous thrombosis, non-union, malunion, or vascular injury.

Serious complications, such as bleeding from the superior gluteal arteries or intra-abdominal injuries, represent rare complications in the literature, which coincide with our findings [27,28]. Based on our study’s findings, we recommend iliosacral screw fixation for cases with bad skin conditions, associated trauma, or circumstances that prevent a prone position. TIFI is a suitable technique for situations characterised by technical difficulties in imaging the fracture and factors that may obstruct the quality of X-rays, such as obesity, the presence of gases, or sacral dysmorphism of anatomical variations.

Limitations and recommendations

The primary limitation of this study is the small number of patients analysed and the short follow-up period. Another limiting factor is the absence of a clear indication regarding the required number of iliosacral screws or ilioilial fixators for effective fixation. 

We propose the need for further randomised controlled trials comparing both methods of managing sacral fractures. We recommend conducting comparative studies assessing the outcomes of sacral fractures treated with single vs. two iliosacral screws and single or double ilioilial fixators.

## Conclusions

Both percutaneous ilioilial fixators and percutaneous iliosacral screw fixations were effective treatment modalities for unstable sacral fractures without spinopelvic dissociation. No differences were noted in terms of perioperative and postoperative assessments, except for the extended incision length in the ilioilial fixator group and the increased number of intraoperative images. Ilioilial fixation is a reliable alternative for sacroiliac screws, especially in cases with challenging intraoperative imaging or abnormal sacral morphology.
